# Contralesional Hemisphere Regulation of Transcranial Magnetic Stimulation-Induced Kinetic Coupling in the Poststroke Lower Limb

**DOI:** 10.3389/fneur.2017.00373

**Published:** 2017-08-07

**Authors:** Andrew Q. Tan, Yasin Y. Dhaher

**Affiliations:** ^1^Northwestern University Interdepartmental Neuroscience, Northwestern University, Chicago, IL, United States; ^2^Searle Center for the Science of Walking, Shirley Ryan AbilityLab, Chicago, IL, United States; ^3^Department of Biomedical Engineering, Northwestern University, Chicago, IL, United States

**Keywords:** transcranial magnetic stimulation, stroke rehabilitation, contralesional hemiphere, lower limb motor control, abnormal synergies, neuromodulation

## Abstract

**Background:**

The neural constraints underlying hemiparetic gait dysfunction are associated with abnormal kinetic outflow and altered muscle synergy structure. Recent evidence from our lab implicates the lesioned hemisphere in mediating the expression of abnormally coupled hip adduction and knee extension synergy, suggesting a role of cortical networks in the regulation of lower limb motor outflow poststroke. The potential contribution of contralesional hemisphere (CON-H) in regulating paretic leg kinetics is unknown. The purpose of this study is to characterize the effect of CON-H activation on aberrant across-joint kinetic coupling of the ipsilateral lower-extremity muscles poststroke.

**Methods:**

Amplitude-matched adductor longus motor-evoked potentials were elicited using single pulse transcranial magnetic stimulation (TMS) of the lesioned (L-H) and CON-Hs during an isometric adductor torque matching task from 11 stroke participants. For 10 control participants, TMS of the contralateral and ipsilateral hemisphere were given during the same task. TMS-induced torques were characterized at the hip and knee joints to determine the differential regulation of abnormal kinetic synergies by each motor cortices. The TMS-induced ratio of knee extension/hip adduction torques was quantified during 40 and 20% of maximum adduction torque.

**Findings:**

For both the 40 and 20% target adduction tasks, we find that contralesional stimulation significantly reduced but did not eliminate the TMS-induced ratio of knee extension/hip adduction torques for the stroke group (*p* = 0.0468, *p* = 0.0396). In contrast, the controls did not present a significantly different TMS-evoked torque following stimulation (*p* = 0.923) of the hemisphere ipsilateral to the test leg.

**Interpretation:**

The reduced expression of abnormal across-joint kinetic coupling suggests that the CON-H may contribute an adaptive role in lower limb control poststroke. Future study of neuromodulation paradigms that leverage adaptive CON-H activation may yield clinically relevant gains in lower limb motor function poststroke.

## Introduction

During walking, abnormal pelvic and lower limb joint motions are apparent in both the sagittal and frontal planes following stroke (1, 2). Evidence of impaired multi-joint coordination poststroke in kinetic output (3) and muscle activation pattern levels (4, 5) suggest that compensatory kinematic strategies are neuromechanical in origin. For example, multi-joint coupling between the hip frontal plane and knee sagittal plane torques were strongly associated with compensatory pelvic movements during gait in persons with stroke (4). While walking recovery interventions that address peripheral kinematics (6, 7) have been evaluated, the neurophysiological mechanisms that centrally mediate abnormal across-joint kinetic coupling remains unclear.

Recent findings from our lab implicates that impaired coordination patterns poststroke are in part cortically driven (8, 9). We observed that single pulse transcranial magnetic stimulation (TMS) of the lesioned hemisphere (L-H) increased the activation of muscles associated with the abnormal across-joint mechanical coupling (3) in the lower limb poststroke. Intriguingly, stimulation of the contralesional hemisphere (CON-H) reduced these activation patterns (8). It remains to be seen if acute modulation in CON-H poststroke adaptively contributes to reductions in abnormal cross-planar kinetic coupling in the lower limb. Given that the ratio of knee extension to hip adduction torque was shown to be a strong predictor of overground gait speed poststroke (10), neuro-rehabilitation paradigms that target synergistic coupling may improve functional recovery. Therefore, a more comprehensive understanding of CON-H regulation of lower limb motor output may lead to the design of more effective approaches for gait rehabilitation.

Converging evidence from upper limb investigations has documented stroke-induced plastic changes in the representations of both L-H (11–13) and CON-H (14, 15). Concurrent with changes in upper limb corticospinal excitability poststroke (16, 17), remapping of sensorimotor cortices was observed in behavioral imaging paradigms (18, 19). While such studies corroborate cortical reorganization poststroke (20), the current range of evidence regarding the role of CON-H in regulating lower limb motor output is sparse. Stroke induced plasticity in CON-H may contribute to abnormal synergistic output in the lower limb yet inferences regarding cortical excitability during gait performance are often constrained to L-H activation (21–23). Other studies highlight locomotor training induced cortical plasticity (24, 25) resulting in localized activation of L-H (26). However, electrophysiological assessments of cortical reorganization of lower limb motor representations following stroke have predominantly focused on changes in corticospinal excitability of specific lower limb muscles (27, 28). Thus, the relationship between CON-H plasticity and specific clinically observed gait deficits poststroke is not well studied. This work addresses this gap, by assessing functionally relevant changes in lower limb motor output resulting from an acute modulation of CON-H.

Toward this end, we further explore the role cortical networks play in the regulation of neural coupling in the lower limb poststroke within the context of previously identified cross-planar coupling of the hip and knee joints. We propose that cortical mechanisms mediating aberrant across-joint coordination may be further clarified in the lower limb by examining the motor evoked torques following TMS. The purpose of this study is to characterize the effect of CON-H activation on aberrant across-joint kinetic coupling of the ipsilateral lower-extremity muscles poststroke. Building upon recently published studies of CON-H regulation of abnormal cortico-excitability coupling (8), we hypothesize that stimulation of the CON-H will reduce the pattern of TMS evoked torque coupling between hip adduction and knee extension in the paretic limb of stroke survivors. Findings from this study will elucidate the role of CON-H in the recovery of the lower limb function following stroke and the potential role of adaptive plasticity to improvements in gait recovery.

## Methods

10 control participants (mean age 37) and 11 stroke participants (mean age 58) with single hemispheric stroke were recruited form the Rehabilitation Institute of Chicago’s Clinical Neuroscience Research Registry (Table 1). All participants gave written informed consent. All experimental procedures were approved by the Institutional Review Board of Northwestern University. Inclusion criteria for stroke participants included: (1) only one monohemipsheric stroke at least 1 year prior to study participation (2) had the ability to perform paretic hip adduction movements during body weight support standing on the non-paretic leg. All stroke participants presented with persistent hemiparesis due to chronic stroke and were classified as community ambulators by their ability to independently walk 10 m without assistances with or without an assistive device (29). Exclusion criteria included severe osteoporosis, contraindications for TMS, history of seizures and medications known to affect central nervous system excitability, history of orthopedic injury or surgery to their lower limbs. Fugl-Meyer scores were collected *post hoc* where available for persons with stroke but were not used as inclusion criteria (Table 1).

**Table 1:** Stroke and control participant characteristics.

Subject	Stroke	Gender	Age	Post (years)	Stroke details	LMFM	Hip adduction (Nm)	Knee extension (Nm)
S1	NA	M	43	8	Right	26/34	37.8	59.4
S2	I	M	64	7	Left insula adjacent frontal, parietal, anterior temporal	NA	21.9	26.9
S3	I	M	60	11	Left	26/34	25.4	42.7
S4	I	M	63	8	Left inferior parietal, anterior temporal	30/34	21.6	23.4
S5	H	M	61	7	Right	NA	20.4	49.6
S6	H	F	53	4	Left basal ganglia	18/34	14	16.6
S7	H	M	56	6	Left thalamic hemorrhage	NA	20	25.4
S8	I	M	50	5	Right internal capsule	NA	18.9	9.2
S9	I	M	67	2	Left middle cerebral artery	16/34	10.2	24.1
S10	H	F	60	2	Right corona radiata, basal ganglia, internal capsule	NA	10.8	10.6
S11	I	M	59	9	Left subcortical	22/34	30.8	15.2
Stroke mean (SD)		M = 9	58 (7)	6 (3)				
Control (*n* = 10)		M = 8	37(11)					

*Maximum voluntary torques for hip adduction and knee extension are listed in the last two columns. Some stroke data was not available due to availability of post hoc assessment*.

*H, hemorrhagic; I, Ischemic; LMFM, lower motor Fugl-Meyer*.

### TMS Protocol

During the torque matching protocol, motor-evoked potentials (MEPs) were elicited in knee extensor and hip adductor muscles of the test limb (paretic limb for stroke, right limb for control) using single pulse TMS according to a protocol previously described (8, 9). Surface electromyography (EMG) with integrated preamplifiers (MA-317, Motion Lab Systems, Baton Rouge, LA, USA) were placed over the following muscles: vastus medialis, vastus lateralis, adductor longus, tibialis anterior. Single pulse TMS was delivered using a Magstim 200 stimulator (Magstim, Whitland, UK) *via* a 110-mm diameter double-cone coil over L-H or CON-H on separate trials.

To map the leg cortical representation hotspot for the test limb ADD, a secured swimming cap placed over the participant’s head was used to demarcate the optimal coil position and orientation for placement consistency. The intersections of the inter-aural and nasion-inion lines were marked as the vertex. The coil position was incrementally adjusted from an initial starting position of 1 cm posterior and 2 cm lateral to vertex (9) to locate the hotspot of the test limb ADD over the lesioned motor cortex (L-H) contralateral to the paretic test limb (right limb for control). Optimal coil position and intensity was mapped in seated position as the lowest intensity of magnetic stimulation required to evoke ADD MEPs of 50 µV in peak-to-peak amplitude in at least three of five consecutive trials (30). The leg cortical representation hotspot for the test limb ADD was similarly mapped for the stroke CON-H or control ipsilateral hemisphere to the test limb. The initial coil position placement was symmetrically placed approximately 1 cm posterior and 2 cm lateral to vertex on the hemisphere ipsilateral to the test limb (9).

Due to the deep medial location of the M1 leg representation within the longitudinal fissure, it is not possible to position the stimulating coil such that either contra- or ipsilateral cortical hemispheres are selectively stimulated by TMS. The response to each stimulus will be therefore be inevitably due to an unknown ratio of bilateral descending activity from *both* hemispheres. To present a normalized comparison of the TMS-induced coupled hip adductor/knee extensor torque, we matched both the TMS-induced ADD torque *and* ADD MEP for each coil placement during an adductor torque matching experiment (below). Achieving equivalent ADD output in MEP *and* torque level for each coil placement provisionally allows the assumption of equal cumulative descending input to the ADD motor neuron pool from both hemispheres. Additionally, the experimental protocol was designed to ensure that the net mechanical outflow of the hip and knee joints were equivalent across coil positions with strict conditions for off axis torque in each trial (below). Therefore, any change from the L-H induced coupled knee extension motor evoked torque output must be due to the activation of additional CON-H neuromotor circuitry (8).

### Torque Target Matching

Isometric hip and knee strength was measured using an experimental paradigm described previously (3, 31). Briefly, participant’s pelvis and paretic limbs were secured to an exoskeleton (Lokomat, Switzerland) instrumented with three 6° of freedom load cells (JR3, Woodland, CA, USA). Maximum voluntary contraction torques (MVC) were recorded in each of the following directions in separate trials: hip flexion, hip extension, hip adduction, hip abduction, knee flexion, knee extension. Next, participants were instructed to match voluntary isometric hip adduction torques at 40 and 20% of maximum voluntary isometric contraction. These contraction levels were chosen to investigate the influence of supraspinal drive on the evoked coupling (31). Control participants only performed the 40% target adduction task. Given that the primary outcome of the study is to quantify abnormal hip adduction and knee extension coupling poststroke, participants used real-time visual feedback to produce primary hip adduction torques while secondary torques at the knee were simultaneously recorded. Subjects were given up to 15 s per trial to volitionally ramp and adjust the joint torque to the target level. A successful trial was considered if the participant was able to match the adduction torque target within +5% of the torque magnitude and hold for a minimum of 200 ms. Additionally, participants were required to keep off axis torques to be less than 5% of MVC for a successful target match trial. Thus, the net knee extension torques was controlled across coil positions such that the mechanical outflow of the paretic leg was identical across trials. All MVC torques and torque matching trials were measured only for the paretic test limb in stroke participants and the right leg of control participants. Load cell signals were filtered offline using a zero-phase, low-pass, fourth-order Butterworth filter with a 50 Hz cutoff frequency. Hip and knee joint torques were calculated from the load cells using static equilibrium equations.

### Target MEP Matching

Stimulator output for CON-H coil placement was increased incrementally in order to match the L-H ADD MEP evoked in the test limb. For each trial, the TMS coil was placed over the hotspot location for either L-H or CON-H hemisphere marked on the swim cap (8). When the participants successfully matched the target adduction torque, the TMS device was digitally triggered to deliver a single pulse using a custom automated software trigger (MATLAB v7.01). The TMS device was not triggered if the participant produced any off-axis torque during the hip adduction or if the target torque magnitude exceeded ±5%. MEPs for each trial were constantly monitored by oscilloscope to ensure that ADD MEP amplitude from both L-H and CON-H coil placements were matched for all trials (8). Stimulator output was accordingly adjusted while secured in the Lokomat to meet this condition and was initially set at 10% higher than the stimulator output used during seated hotspot mapping. Additionally, given the sensitivity of applied magnetic field to head orientation, a custom-built coil holder was used to defray slippage of the coil as well as Velcro adhesives securing the coil location. As a second control condition, all TMS-evoked adductor torques form the test limb were matched across trials between L-H and CON-H coil placements. The magnitude of the induced torque was not required to be a target percentage of the baseline torque. Not every trial achieved the control conditions of matched TMS evoked ADD torque and ADD MEP. Thus, 10–30 trials were performed for each hemisphere and for each torque matching condition with adequate rest periods between trials. Stimulation block of 10 trials targeting one hemisphere and one target torque level were completed in series to minimize coil movement. A complete stimulation set for one target torque level for each hemisphere were completed before resetting the experiment to the next target torque level.

## Data Analysis

### Motor-Evoked Potentials

To identify the onset latency of the TMS-induced MEP in the EMG of each muscle, the EMG signal during the target matching task was filtered with an 8th order Butterworth, low-pass, zero-phase digital filter with a 220 Hz cutoff frequency. The filtered EMG signals were rectified and smoothed using the same digital filter with a 20 Hz cutoff frequency. MEP onset latencies were computed for each primary muscle in each participant from the ensemble of successful hip adduction trials. A pre-TMS background level of EMG activity was defined by calculating the mean and SD of activity within a 100 ms window prior to a recorded TMS trigger pulse (32). An extended 80 ms window following the TMS trigger pulse was used to search for the onset of MEPs (27) that were clear and distinguishable from background activation (17). MEP onset was identified when five consecutive points in the EMG trace were above three SDs of the mean pre-TMS EMG activity.

### TMS-Induced Torque Response

**Figure 1:**
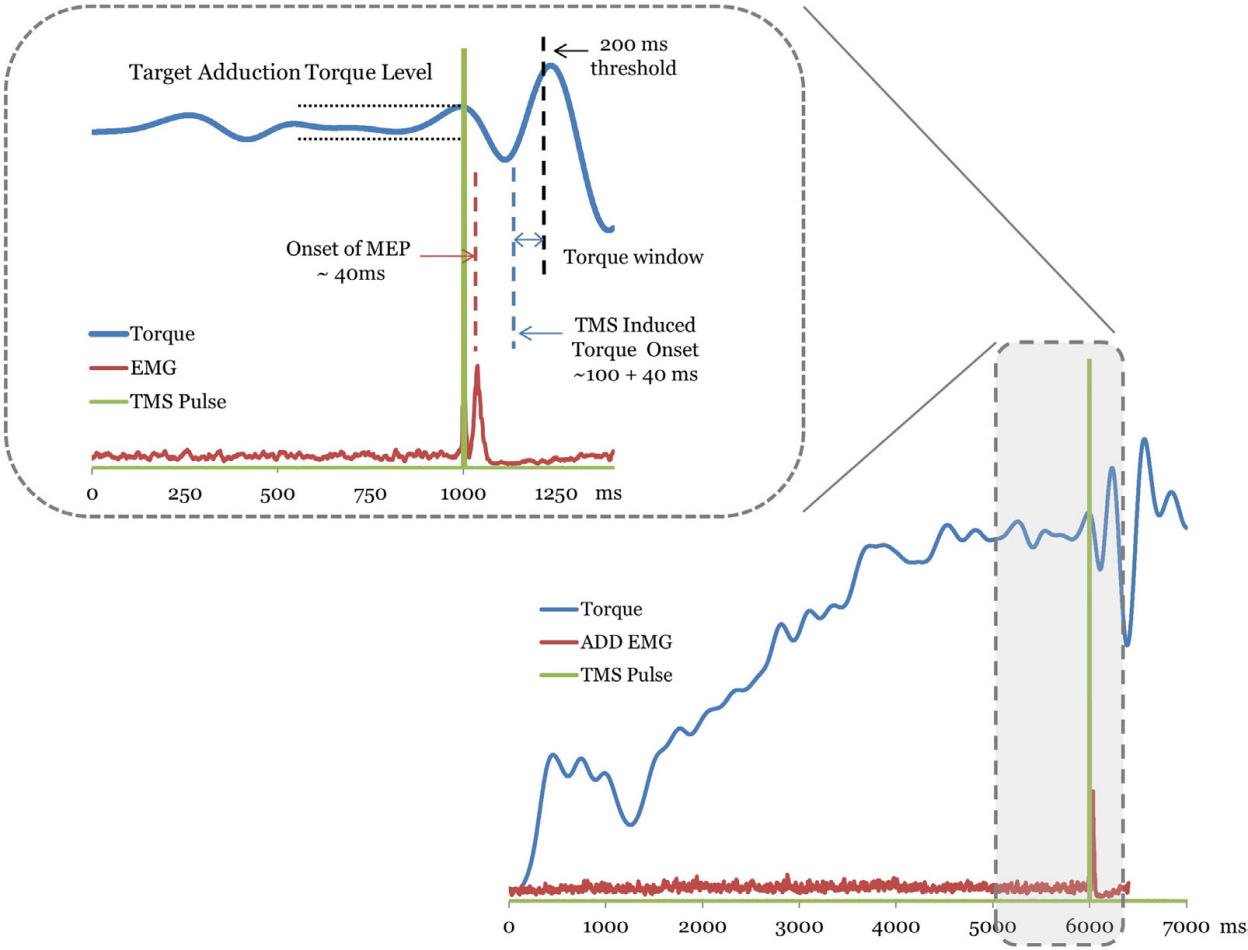
A trace of the torque, electromyography (EMG), and transcranial magnetic stimulation (TMS) signal from a representative stroke subject trial. The TMS stimulation onset is plotted as a square pulse. Motor-evoked potentials (MEPs) are labeled with the dashed line over the EMG trace. The plotted EMG is the rectified raw signal. For this trial, the MEPs for the adductor muscle occurred ~40 ms following the TMS pulse. The TMS-evoked torque onset window begins ~100 ms latency following the MEP. The inset magnifies the region of the torque trace used to determine the TMS-induced torque. Muscle torques greater than 200 ms latency (dashed line) following MEP were considered volitionally produced (see Methods).

Once the MEP onset is identified, the magnitude of the TMS-induced torque was calculated by identifying the peak torque (Figure 1). A window beginning 100 ms following the MEP onset latency was used to search for the TMS-induced torque. Voluntary contraction times of knee have been reported to fall in the range of 215–220 ms and triggered reactions occur at about 175 ms (33, 34) following mechanically induced activity at the human knee. Torque responses exceeding a conservative threshold of 200 ms were considered to be longer latency volitional reactions and were not considered for the analysis (Figure 1). The incremental torque in each secondary degree of freedom (knee flexion/extension) resulting from TMS was normalized to the MVC of the corresponding secondary torque measure. The difference between the TMS-induced torque and the level of voluntary torque at the target hip adduction was calculated for the 20 and 40% MVC target levels. The incremental torque vector was used to examine the effects of hemisphere and target level on the TMS-induced across-joint coupling poststroke (Figure 1). Pre-stimulus background EMG activation of ADD was matched across trials for all hemispheres through the torque matching paradigm.

### Statistical Analysis

Independent samples *t*-test were used to evaluate differences in the average maximum voluntary torque contractions at the hip and knee joints between the control and stroke groups. To evaluate statistically significant differences in the TMS-induced knee extension torque following coil placements, a two-way mixed design ANOVA was performed with group (stroke vs control) as the between subjects factor and coil placement (L-H vs CON-H) as the within subjects factor. To further explore the effect of activation level on the TMS-induced knee extension torque in persons with stroke, a two-way, repeated measures ANOVA was used to evaluate the within subjects factors of torque level (40 vs 20%) and coil placement (L-H vs CON-H) in the stroke group only. Post-hoc *t*-tests with a Bonferroni adjustment for multiple comparisons were used to evaluate any statistically significant differences between the TMS-evoked torques resulting from L-H and CON-H coil placement within each group. Finally, TMS-evoked knee extension/hip adduction torque ratios were evaluated with paired *t*-tests across coil placements. A *post hoc* analysis was performed to validate if all successful trials included in the group averages achieved statistically equivalent ADD background activations and matched TMS-evoked ADD torques across coil placements.

## Results

### Maximum Voluntary Torques Reduced in Persons with Stroke

Maximum voluntary contractions produced at the hip joint by stroke participants were smaller than those of control participants in target directions. The maximum knee extension and hip adduction torque magnitude for each stroke participant are listed in Table 1. Specifically, the stroke group’s hip flexion torque was significantly smaller than the controls (*p* = 0.0365). However, while hip frontal plane torques were smaller in magnitude, they were not significantly different between groups for hip adduction (*p* = 0.386) and hip abduction (*p* = 0.489). Sagittal plane knee torques produced by stroke participants were significantly smaller than controls in both knee flexion (*p* = 0.0484) and knee extension (*p* = 0.00273) directions.

### TMS-Induced Adduction Torques and MEP Were Matched across Coil Placements

**Figure 2:**
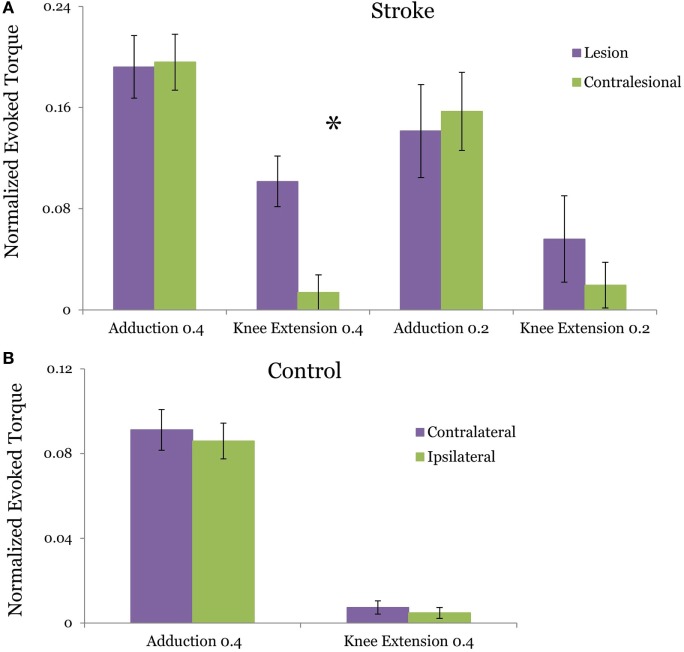
Group averages of the transcranial magnetic stimulation (TMS)-evoked hip adduction and knee extension torque resulting from lesion and contralesional hemisphere (CON-H) coil placement. The plotted torques occur during a target hip adduction task of 40 and 20% of maximum voluntary torque. Purple bars indicate lesion hemisphere coil placement (contralateral to test limb in control) while green bars indicate CON-H coil placement (ipsilateral to test limb in control). All torques are normalized to maximum voluntary production for each direction. Error bars indicate SEM. The evoked adduction torques and knee extension torques are plotted for stroke in **(A)** and for control in **(B)**. In stroke, a significant reduction in the TMS-evoked knee extension torque was observed following CON-H coil placement (*p* = 0.0126) during the 40% task but was marginal during the 20% task (*p* = 0.0923). No significant reduction in the TMS-evoked knee extension torque (*p* = 0.544) was observed in controls following ipsilateral hemisphere coil placement. In both groups, we successfully matched the evoked adduction torque following coil placement with no significant difference between the motor-evoked adduction torques following coil shift (*p* = 0.923 for stroke, *p* = 0.439 for control).

In the stroke group, the TMS evoked adduction torque elicited by TMS of L-H and CON-H hemispheres were not significantly different from each other at both the 40% (*p* = 0.923) (Figure 2A) and 20% target matching levels (*p* = 0.3125). Similarly, for control participants, L-H and CON-H TMS-evoked adduction torques were not significantly different from each other during the 40% matching task (*p* = 0.439) (Figure 2B). To achieve equivalence in TMS-evoked torque and MEP, stimulation intensity for CON-H coil placement was increased for all subjects relative to L-H stimulation intensities. To further validate the control condition of matched, ADD background torque activation for each coil placement, we found that the pre-stimulus adduction torques were not significantly different from each other for controls (*p* = 0.936) and for stroke participants (*p* = 0.933) at the 40% matching task. Together, these confirm that participants successfully matched both the pre TMS background activation of ADD and that the TMS-induced adduction torques were equivalent following L-H and CONH coil placements.

### TMS-Evoked Knee Extension Torque Reduced following CON-H Stimulation

Figure 2 plots the TMS motor-evoked knee extension torques elicited following TMS of the lesioned and CON-H in stroke participants normalized to MVC. Figure 2A plots the induced torque during 40 and 20% MVC adduction target for stroke participants, while Figure 2B plots the induced torque for controls. We observed a reduction in the TMS-induced knee extension torque in 10 out of 11 stroke participants during CON-H coil placement. The two-way mixed design ANOVA results reveal a significant main effect of group (*F* = 4.903, *p* = 0.033) and coil placement (*F* = 4.497, *p* = 0.041) for the TMS-evoked knee extension toque. A significant interaction effect between group and hemisphere was found (*F* = 4.44, *p* = 0.042). These moderate levels of significance are primarily driven by the expected absence in evoked TMS knee extension torque in controls across both coil placement. These results therefore indicate that only stroke participants produced coupled knee extension torques for each coil position and that CONH stimulation significantly reduced this evoked torque.

The two-way repeated measures ANOVA for the stroke group revealed a non-significant (*F* = 0.115, *p* = 0.737) difference between the magnitude of the TMS-evoked knee extension torque during 40 and 20% MVC adduction level. However, a significant effect of coil placement was found (*F* = 5.637, *P* = 0.023). A non-significant interaction effect between torque level and coil placement (*F* = 0.252, *p* = 0.619) in the stroke group. Due to the expected lack of difference in evoked knee extension torques for able-bodied subjects between coil positions (Figure 2B, *p* = 0.544), *post hoc t*-tests with Bonferroni adjustment for multiple comparisons were used to further compare knee extension torques LH vs CON-H coil placements at each effort level in persons with stroke. For the 40% adduction torque matching task, we observed a significant reduction in the knee extension torque following CON-H TMS (*p* = 0.0126). A corresponding reduction in knee extension torque was observed following CON-H TMS in the 20% adduction torque reduction task but was not significant (*p* = 0.0923).

### TMS-Induced Knee Extension/Hip Adduction Ratio Reduced following CON-H Stimulation

**Figure 3:**
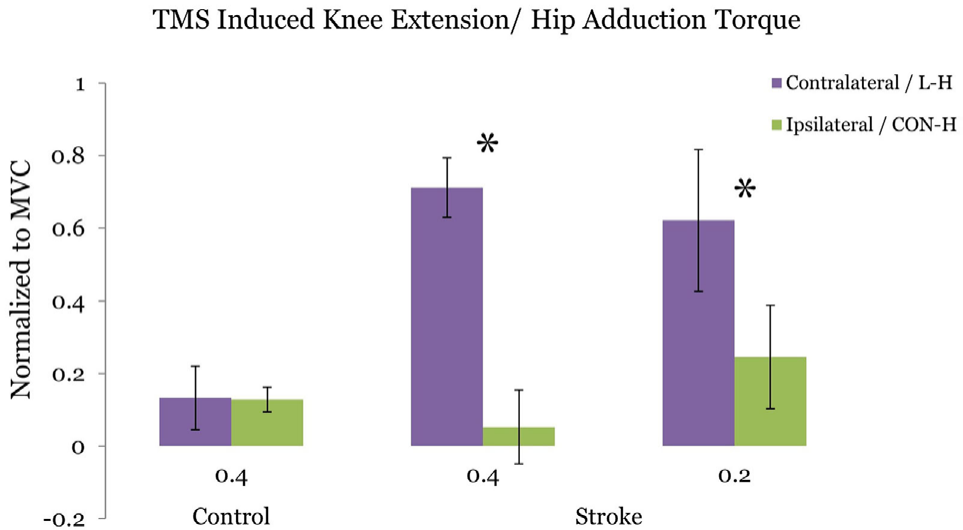
Ratio of the transcranial magnetic stimulation (TMS) induced hip adduction/knee extension torques. Purple bars indicate lesion hemisphere coil placement (contralateral to test limb in control), while green bars indicate contralesional hemisphere (CON-H) coil placement (ipsilateral to test limb in control). Group averages are plotted. The plotted torques are normalized to maximum voluntary torque production for each direction. Error bars indicate SEM. No significant reduction in the TMS-induced torque ratio is observed for the control group at the 40% target adduction level following ipsilateral hemisphere coil placement (*p* = 0.923). Asterisks represent a significant reduction in the TMS-induced torque ratio following CON-H coil placement at both the 40% (*p* = 0.0468) and the 20% (*p* = 0.0396) target adduction level for the stroke group.

Figure 3 plots the ratio of TMS-evoked knee extension/hip adduction torque at the 40 and 20% target-matching condition, respectively. For stroke participants, we observe a significant reduction in TMS evoked knee extension/hip adduction toque ratio at the 40% adduction task (*p* = 0.0468) following CON-H coil placement. At 20% target adduction, we also observe a significant reduction in the knee extension/hip adduction motor-evoked torques (*p* = 0.0396) in the stroke group. In contrast with stroke participants, controls did not present significantly different TMS-evoked knee extension/hip adduction ratios following CON-H coil placement (*p* = 0.983) at 40% target adduction (Figure 3).

## Discussion

The current study characterized differences in the contribution of lesioned and contralesional motor cortices to abnormal across-joint kinetic coupling poststroke between hip adduction and knee extension. We quantified single pulse TMS-evoked torques in the lower limb resulting from either L-H or CON-H stimulation using a torque matching paradigm. After controlling for ADD output across coil placement location, we found that CON-H stimulation significantly reduced the expression of abnormally coupled knee extension torques in individuals with stroke during hip adduction whereas stimulation of L-H increased knee extension coupling. Stimulation of either L-H or CON-H in the control group did not evoke knee extension torques during hip adduction. Furthermore, this reduction in abnormal across-joint coupling in persons with stroke was observed at both the 40 and 20% torque matching levels.

These findings are congruent with previous studies that identify impaired multi-joint coordination in the poststroke lower limb as a potential source of gait dysfunction (31). Abnormal synergistic coupling between the hip frontal and knee sagittal planes (3) have been shown to be negatively related to poststroke gait speed (10). Thus, volitional motor impairments resulting from abnormal cross-planar torque and muscle activation coupling (9) may influence clinically observed frontal plane movements (1). The current observations link prior neurophysiological evidence of acute CON-H regulation of muscle activation (8) with a functionally relevant paretic leg mechanical output. Together, these results suggest that CON-H may mediate an adaptive role in paretic lower limb control poststroke. Rehabilitation paradigms directed at reducing aberrant synergistic coupling may lead to improvements in gait speed and kinematics.

### Induced Torque vs MEP

Reductions in the coupled knee extension torque confirms the pattern of reduced knee extensor MEPs following CONH simulation (8). While a comprehensive characterization of MEP amplitudes and latencies for multiple leg muscles have been previously reported (8), the evoked torques observed here provides additional insight regarding the functional implications of these MEP patterns. Associations between the reduced complexity of lower limb muscle excitation patterns poststroke (35) with impairments in specific biomechanical subtasks emphasize the importance of characterizing functionally relevant mechanics. The TMS-induced reduction of knee extensor torques revealed an adaptive cortical role in regulating abnormal mechanical outflow across the hip and knee joints. The pattern of aberrant joint torque reduction encompasses a cumulative sum of multiple muscle twitches acting upon multiple axis from a single TMS pulse. The net mechanical output therefore includes muscle twitches that contribute to opposing knee flexion torques from the same TMS volley. The observation of a net knee extension torque across coil placements indicates that muscle twitches contributing to knee flexion, such as the hamstrings, have a smaller contribution to the induced torque for each coil placement. We did not observe a significant increase in hamstring MEP’s during CON-H placement in Ref. (8), suggesting that reductions in KE torque observed may be attributed to the differential activation of muscles that produce knee extension but not knee flexion. Thus, the changes in the net joint torque confirm an abnormal mechanical outflow associated with the previously reported pattern of MEP coupling.

### Functional Reduction in Impaired Coordination

The current findings further support the notion of centrally mediated impaired multi-joint coordination in the poststroke paretic limb. The reduction of the TMS evoked secondary torques at both the 20 and 40% levels following CON-H coil placement suggests a cortical role in regulating characteristic kinetic outflow patterns poststroke. Both background activation as controlled by pre-stimulus adductor EMG, and effort as measured by percentage of MVC ADD torque, did not affect the pattern of torque coupling. This finding is consistent with previous studies from our lab that characterized altered multi-joint kinetic signatures that are invariant with both posture and level of supraspinal drive (31). Interestingly, the ratio of the TMS-evoked knee extension/hip adduction torques are smaller in magnitude to that previously reported volitional secondary/primary torque production under similar isometric conditions (4). The ratio of paretic knee extension to hip adduction was 1.2 in the previous study compared to the current findings of 0.71 and 0.62 at the 40 and 20% matching tasks, respectively (Figure 3). The reduced evoked torque values observed here reflects the net magnitude of multiple muscle twitches above a significant tonic contraction of 20 or 40% MVC. Thus, the TMS-induced torque may therefore be more analogous to electrically evoked muscle twitches superimposed during MVC contractions used to quantify central activation deficits (36) in persons with stroke and incomplete spinal cord injury. Given that both the volitional and TMS-evoked responses drive neural coupling from L-H activation, it seems likely that the neural substrates underpinning discrete volitional control objectives may correspond with the cortical networks activated by single pulse TMS.

While correlations between isometrically evaluated coupling have been made to deficits in gait speed (4), only conditional inferences regarding CON-H regulation of walking can be made. Indeed, walking is fundamentally dynamic, involving complex spinal level modulation (37) of parallel descending drive pathways not discerned by with the current paradigm. Any cortical regulation of the kinetic synergies observed here is further shaped by spinal network reorganization post-injury that integrates both central pattern generators and afferent feedback during locomotion.

### Lesioned Hemisphere Stimulation Induces Abnormal Joint Torque Coupling

A confound shared by the present study and other published studies involving TMS of CON-H is that the observed electrophysiological responses may be primarily attributed to L-H activation by current spread from a CON-H coil position. It may be argued that an ipsilateral coil position (1) concurrently activated contralateral motor cortex due to the close proximity of the leg motor representations and (2) this concurrent activation is the primary driver of the reduced knee extension. While the lesioned hemisphere is certainly activated during CON-H placement, changes in L-H activation alone may insufficiently account for the present results. This intuition is based upon poststroke L-H stimulation results indicating that stroke induced changes within L-H only contributed to increases in cross-planar coupling between hip adductors and knee extensors but not reductions (8, 9). Thus, if differences in L-H activation alone is *primarily* driving the results, one would expect the ratio of knee extension/adduction torque to be similar or greater for CON-H coil placement since increases in stimulation were used to match ADD MEP in all participants (see Methods). Therefore, both the decrease in the induced knee extension torque and knee extensor muscle MEP (8) provide evidence that additional CON-H activation reduced the cross-planar coupling at both levels.

### Pre-stimulus Kinetic Output Was Functionally Equivalent across Trials

The experimental paradigm was designed to ensure that the pre-stimulus neuromotor state was tuned to produce approximately similar functional output between groups and coil positions. For example, descending drive to the motoneuron pools for the ADD was equally activated for each trial, regardless of coil placement location. Statistical analysis confirmed that both the evoked adductor torque and MEP was not significantly different following coil shift in both groups. A key limitation of the current paradigm was that stimulation of the knee extensor representation was not directly controlled across coil placements. However, one crucial control condition achieved here required that the participant produce no secondary off-axis torques during the target adductor matching to trigger the TMS (9). Therefore, volitional drive to knee extensors was approximately equivalent for each trial as any off axis torques were less than 5% MVC. Since any concomitant coactivation of knee extensor muscles that produced off axis torque was controlled, the contribution of knee extensor activation differences to the induced torque was minimized but not eliminated. Similarly, differences in MVC strength between participants for each joint (Table 1) may indicate the amount of residual, volitional drive to each joint poststroke. Although there were a wide range of MVC strength between participants, the significant reduction in the normalized TMS-evoked knee extension/hip adduction suggests that variations in individual knee extension or hip adduction strength may play a minor role.

The observation that controls did not show changes in the evoked knee extension torques helps to clarify if the observed coupling exists merely by the location of the knee and adductor representation in cortex. Specifically, knee extension torques did not change after CON-H stimulation in control subjects, consistent with our previous finding of no MEP amplitude reduction of individual knee extensor muscles across coil positions (8). The control results therefore support the idea that in the absence of any stroke-induced changes in each cortex, biasing the stimulation of each cortex by coil placement may not necessarily impose any neural coupling between hip adductors and knee extensors.

### CON-H May Regulate Functional Lower Limb Output Poststroke

An important limitation is that the current results cannot identify specific plastic changes in CON-H that mediated the reduction in abnormal coupling. The current paradigm was not designed to apportion the effect of each hemisphere. Rather the present evidence and previous MEP characterization (8) supports the interpretation that biasing CON-H activation functionally reduced the expression of aberrant cross-planar torque coupling. However, the current results cannot dissociate between the many compelling mechanisms of L-H reorganization that may explain the L-H-induced coupling such as the merged representations of hip adductors and knee extensors following stroke. In our view, it may not be necessary to know the specific changes in L-H reorganization that induced abnormal coupling to determine if CON-H activation had an effect. While stroke-induced changes in L-H may clarify our L-H stimulation results, they incompletely account for reductions in coupling following CON-H stimulation. We speculate that putative mechanisms of ipsilateral corticomotor activation may not be mutually exclusive with any reduced differentiation of lower-extremity representations in L-H but is beyond the scope of the current paradigm.

### Contralesional Pathways for Lower Limb Functional Recovery

The contributions of ipsilateral and contralateral pathways to lower limb kinetic synergies have not been examined previously in stroke survivors. The putative functional role of ipsilateral pathways in upper limb recovery following stroke have been investigated by several groups (14, 38, 39). However, increases in ipsilateral descending motor pathways poststroke have been associated with poor motor recovery, indicating a limited role of ipsilateral pathways in upper limb motor recovery (40, 41). In contrast with our findings, Schwerin et al. reported that increased excitability of ipsilateral pathways to proximal upper limb may contribute to the expression of abnormal elbow/shoulder coupling (42). However, our data implicate a role of ipsilateral pathway activity that is functionally advantageous. These equivocal results may be attributed to fundamental differences in the neurophysiological substrates underlying upper and lower limb control (37). Spinal locomotor circuitry further modulates the expression of abnormal coupling during walking although the interaction was not examined here. Given that all stroke participants tested were community ambulators and able to consistently perform a demanding torque matching task, the current study most likely reflects a subset of stroke survivors in which lower limb MEPs were consistently attainable. Therefore, it remains inconclusive from the current data if the reduction in inter-joint coupling reflects the recruitment of ipsilateral corticospinal pathways (43).

Specific interhemispheric models of poststroke plasticity for the upper limb propose neuromodulation paradigms to rebalance the abnormally high inhibitory drive from CON-H (44, 45). It remains to be seen if neuromodulation of underlying CON-H connectivity may adaptively reduce abnormal coupling in muscle activation (46) and the kinetics of the paretic limb. As clinically relevant improvements in gait resulting from reduced coupling may be gained, leveraging non-invasive stimulation methods such as repetitive TMS (rTMS) may further elucidate CON-H contributions for lower limb recovery. Intriguingly, rTMS applied over the leg area of motor cortex of CON-H have been shown to be effective in modulating gross gait metrics after stroke (47, 48). Thus, CON-H coil placement may have sufficiently bias the activation of CON-H networks that reduced the aberrant synergy. Indeed, the feasibility of modulating one lower limb cortex in humans poststroke has been previously demonstrated using paired associative stimulation (22, 28, 49) and tDCS (50, 51). Neuromodulation strategies directed at asymmetries in interhemispheric competition may therefore play a significant role in shaping lower limb motor output poststroke and merits further investigation.

## Conclusion

In summary, the current study provides evidence that cortical networks acutely activated by CON-H TMS may be involved in regulating the expression of abnormal across-joint torque coupling in the paretic lower limb. Contrary to evidence of the maladaptive role of CON-H in the upper limb poststroke, analysis of the TMS-induced torques suggests that plasticity in the neural substrates underlying volitional lower limb control may yield a functionally relevant output for gait recovery. Further study is needed to clarify if sustained changes in CON-H excitability *via* neuromodulation paradigms may potentially shape lower limb function following stroke. Future work relates how CON-H regulation of kinetic outflow corresponds with alterations in the composition of muscle activation patterns available for locomotion.

## Ethics Statement

Study participants were recruited form the Rehabilitation Institute of Chicago’s Clinical Neuroscience Research Registry (Table 1). All participants gave written informed consent according to the Declaration of Helsinki. All experimental procedures were approved by the Northwestern University Institutional Review Board.

## Author Contributions

AT contributed to the study design, participant recruitment, data collection, analyses, and interpretation. AT also performed statistical analysis, literature search, figures, and contributed to manuscript revisions. YD was the principal investigator for this study. He contributed to the study concept and design, data analyses and interpretation, literature search, figures, and manuscript revisions.

## Conflict of Interest Statement

The authors declare that the research was conducted in the absence of any commercial or financial relationships that could be construed as a potential conflict of interest.
